# Artificial Intelligence (AI) as a Cognitive Function Digital Biomarker: Analyzing Speech in Older Individuals

**DOI:** 10.1093/geroni/igaf122.1757

**Published:** 2025-12-31

**Authors:** Catherine Diaz-Asper, Mahederemariam Dagne, Elizabeth Terhune, Erin Staker, Patricia Heyn

**Affiliations:** Marymount University, Arlington, Virginia, United States; Marymount University, Arlington, Virginia, United States; Marymount University, Arlington, Virginia, United States; Center for Optimal Aging, Arlington, Virginia, United States; Marymount University, Arlington, Virginia, United States

## Abstract

Artificial intelligence (AI) technologies designed to analyze speech have demonstrated considerable potential as screening tools for Alzheimer’s disease (AD), enabling the detection of subtle alterations in the content and structure of natural speech. The most common AD markers in speech include word finding difficulties, reduced coherence, and longer pauses between words and sentences. Natural Language Processing (NLP) and acoustic analysis can identify subtle linguistic and structural changes associated with cognitive decline. However, most AI models are trained on English-speaking populations, limiting their global applicability. In fact, most NLP research focuses on about 20 of the 7,000 languages spoken worldwide, ignoring the roughly billion speakers who speak other, low resource languages —languages that are underrepresented, under-researched, and under-computerized. In the United States alone, over nine million older adults speak a language other than English at home, potentially limiting their access to speech-based cognitive screening tools. Additionally, due to the limitation in current datasets most NLP platforms do not account for different dialects and accents, further limiting their uses even to English speakers with intonations that differ from the reference speaking style. To reduce disparities and ensure AI models can be applicable to more individuals, it is imperative to prioritize research into low-resource languages in the development of AI-driven screening technologies. Thus, this research focuses on developing inclusive AI-driven cognitive screening tools by integrating diverse linguistic datasets and analyzing speech patterns among non-English-speaking older adults. Findings will contribute to equitable AI applications in dementia screening and cognitive health research.

